# Selenium in the Prevention of Anthracycline-Induced Cardiac Toxicity in Children with Cancer

**DOI:** 10.1155/2012/651630

**Published:** 2012-10-16

**Authors:** Nurdan Tacyildiz, Derya Ozyoruk, Guzin Ozelci Kavas, Gulsan Yavuz, Emel Unal, Handan Dincaslan, Semra Atalay, Tayfun Ucar, Aydan Ikinciogullari, Beyza Doganay, Gulsah Oktay, Ayhan Cavdar, Omer Kucuk

**Affiliations:** ^1^Department of Pediatric Oncology, Medical School, Ankara University, 06590 Ankara, Turkey; ^2^Department of Physiopathology, Medical School, Ankara University, 06590 Ankara, Turkey; ^3^Department of Pediatric Cardiology, Medical School, Ankara University, 06590 Ankara, Turkey; ^4^Department of Pediatric Immunology, Medical School, Ankara University, 06590 Ankara, Turkey; ^5^Department of Statistics, Medical School, Ankara University, 06590 Ankara, Turkey; ^6^Turkish Academy of Sciences, Cankaya, 06550 Ankara, Turkey; ^7^Winship Cancer Institute, Emory University, Atlanta, GA 30322, USA

## Abstract

High cumulative doses of anthracyclines (300–500 mg/m^2^) used in the treatment of children with cancer may result in cardiotoxicity, a major long-term adverse effect that limits clinical usefulness of this class of chemotherapeutic agents. We assessed anthracycline-induced cardiotoxicity by measuring Pro-BNP levels and echocardiographic (ECHO) findings and investigated potential protective effect of selenium (Se) supplementation in a group of pediatric cancer patients. Plasma level of Pro-BNP was measured, and ECHO was performed in 67 patients (45 boys, 22 girls; ages 2–18 years; median age 12 years) after they completed anthracycline-containing chemotherapy. Serum Se level was measured in 37 patients. Eleven patients had high Pro-BNP levels and/or cardiac failure with Pro-BNP levels of 10–8,022 pg/mL (median 226.3 pg/mL; laboratory normal level is less than 120 pg/mL). Serum Se levels were low (20–129 mcg/L, median 62 mcg/L) in ten of these eleven patients. Eight of 10 patients with low Se and high Pro-BNP levels were supplemented with Se 100 mcg/day for a period of 4–33 months (median 6 months) which resulted in improvement in Pro-BNP and/or ECHO findings. These results suggest that Se supplementation may have a role in protection against anthracycline-induced cardiac toxicity.

## 1. Introduction

High cumulative doses of anthracyclines (300–500 mg/m^2^) are frequently administered to children with cancer. Cardiac toxicity is a serious adverse effect that limits the therapeutic potential of anthracyclines and threatens the cardiac function of pediatric cancer patients leading to debilitating long-term effects resulting in poor quality of life in cancer survivors [1–5]. This is particularly devastating in children who are cured of their cancer because they have to endure the debilitating cardiac dysfunction for the rest of their lives with limited exercise capacity which may also lead to other chronic illnesses.

B-type-natriuretic peptide (BNP) is a polypeptide hormone predominantly released from the cardiac ventricles in response to volume expansion and pressure overload. BNP is found in the circulation as BNP-32 and the NH_2_-terminal portion of ProBNP (Nt-proBNP). BNP levels are elevated in patients with left ventricular systolic dysfunction and correlate with the severity of symptoms and prognosis [6–14]. Measuring serum Pro-BNP levels is a reliable way to monitor the cardiac function of patients receiving cardiotoxic drugs such as anthracyclines.

Selenium (Se) is a trace element distributed in a small amount in the soil and certain foods. It is an important antioxidant, and its absence has been associated with cardiomyopathy in people living in areas with poor levels of soil Se. The concentration of Se in grain varies based on the soil content. Dietary Se is found in meat and seafood. It is a cofactor for glutathione peroxidase which catalyzes the reduction of hydrogen peroxide using glutathione. It is an essential element to remove free radicals from the body and to prevent oxidative tissue damage [15–19]. Se supplementation could potentially prevent cardiac toxicity of anthracyclines [16–20].

In this study, we assessed anthracycline-induced cardiotoxicity by measuring Pro-BNP levels and echocardiographic (ECHO) findings, and we investigated the potential protective effect of Se supplementation in a group of children with high Pro-BNP levels and/or cardiac dysfunction. 

## 2. Patients and Methods 

Plasma level of Pro-BNP was measured, and echocardiography (ECHO) was performed in 67 pediatric cancer patients (45 boys and 22 girls, ages between 2 and 18 years, median age 12 years) with a variety of tumors (leukemias, lymphomas, solid tumors) after completing anthracycline-containing treatment. Serum Se levels were measured in 37 patients. Sera were stored at −20 degrees centigrade until selenium levels were measured with atomic absorption method. Patients with low level of Se were supplemented with Se (100 mcg/day). 

## 3. Statistical Analysis

Statistical analysis was performed using SPSS (Version 15.0) software package. Comparisons between the groups were done using Mann-Whitney  *U*  test, Wilcoxon sign test, and Fisher's exact test. Levels of statistical significance were set at a  *P*  value < 0.05. The results were expressed as range (minimum and maximum) and median.

## 4. Results 

In eleven patients who had high Pro-BNP levels and/or cardiac failure Pro-BNP levels ranged between 10 and 8022 pg/mL with a median of 226.3 pg/mL (normal < 120 pg/mL). Fifty-six patients had normal Pro-BNP levels (8.2–119.6 pg/mL, median 32.4 pg/mL). As seen in Table 1, the difference in levels of Pro-BNP between these two groups was significant (*P* < 0.001). Serum Se levels were low in 10 of these 11 patients with high Pro-BNP levels and/or cardiac failure (20–129 mcg/L, median 62 mcg/L). Twenty-six of 56 patients with normal Pro-BNP levels were also investigated for Se levels (51.3–150 mcg/L, median 99.4 mcg/L). There was a significant difference between Se levels of patients in high Pro-BNP and normal Pro-BNP groups (*P* < 0.001) (Table 1). 

Abnormal ECHO findings were observed in 7 of 11 (63.6%) patients with high Pro-BNP levels and/or cardiac failure group. Only 1 (3.8%) of 26 patients with normal Pro-BNP levels had abnormal ECHO finding. A patient with normal pro-BNP and low Se level died in 1 month because of progressive disease with respiratory failure and cardiac failure. The probability of having abnormal ECHO findings was significantly higher in patients with high Pro-BNP compared to those with normal Pro-BNP (*P* < 0.001) (Table 2). Eight of 11 patients with low Se and high Pro-BNP levels were supplemented with Se 100 mcg per day for 4–33 months (median 6 months). Three of 8 patients had cardiac failure according to ECHO and were supplemented with Se in addition to digoxin and ACE inhibitors. All 3 patients were doing well with normal ECHO findings and normal Pro-BNP levels after a follow-up periods of 33, 14, and 5 months. Five patients, 3 with normal ECHO and 2 with diastolic dysfunction (one with low Pro-BNP level, other with high Pro-BNP level) also, were supplemented with selenium (100 mcg per day). One patient who had diastolic dysfunction with normal Pro-BNP did well with Se supplementation with normalization of ECHO findings, but she later died due to progression of her cancer. Another patient with diastolic dysfunction as well as 3 patients with normal ECHO had normal Se and Pro-BNP levels after 4–6 months of Se supplementation. Only 3 patients were not supplemented with Se in the high Pro-BNP and/or cardiac failure group, because one of them had normal Se level, the second one died with progressive disease in a very short period of time, and the third one had Pro-BNP level within normal limits after the removal of intracardiac tumor thrombus with open heart surgery (Table 3). In Se-supplemented group, supplementation period was between 4 and 33 months (median 6 months). Before supplementation, Pro-BNP levels were between 10 and 843 pg/mL (median 175 pg/mL). After supplementation, Pro-BNP levels were 2–536 pg/mL (median 73.5 pg/mL) which were significantly lower than pretreatment levels (*P* = 0.018). Pretreatment Se levels were between 20 and 83 mcg/L (median 57 mcg/L). After supplementation Se levels were 65–109 mcg/L (median 103 mcg/L) which were significantly higher than presupplementation level (*P* = 0.028) (Table 4). After achieving normal Se and Pro-BNP levels, Se supplementation was discontinued. During follow-up period with no Se supplementation, 2–6 months after supplementation repeat measurements of Se levels were 75–106 mcg/L (median 83 mcg/L), and Pro-BNP levels were 10–123.5 pg/mL (median 106.5 pg/mL), which were lower for Se (*P* = 0.068) and higher for Pro-BNP (*P* = 0.109) compared to Se-supplemented period (Table 4).

## 5. Discussion

The main long-term toxicity of anthracyclines is cardiac dysfunction associated with their chronic and/or high-dose administration. Severe cardiomyopathy and congestive heart failure may develop any time after the completion of the treatment. The precise pathogenesis of anthracycline-induced cardiotoxicity is still uncertain, and it is likely to be multifactorial in origin. Nevertheless, pivotal role is attributed to the iron-catalyzed intramyocardial production of reactive oxygen species (ROS), which cause damage of various targets in the myocardial cells [1–5]. Probrain natriuretic peptide (Pro-BNP) is released by cardiac cells, and serum levels are elevated even before the development of overt cardiac distress symptoms related to impairment of left ventricular systolic or diastolic function leading to increased left ventricular wall stretch. Recent studies have also suggested that ischemia itself may promote release of BNP [7, 20–24]. In the present study, we evaluated cardiotoxicity in 67 pediatric patients with cancer (leukemia, lymphoma, and solid tumor) after they completed treatment with anthracycline-containing regimens. We also evaluated Se levels and the effects of Se supplementation with regard to cardiotoxicity because previous studies with Keshan disease (KD) suggested potential protective role of Se for cardiac dysfunction observed in Se deficiency.

KD, a potentially fatal form of cardiomyopathy, first found in Keshan county, northeast China, is one of the most harmful endemic diseases. The disease is characterized by multifocal myocardial necrosis and fibrosis and leads to congestive heart failure and cardiogenic shock. Although the exact etiology of KD is unclear, Se deficiency is a major contributing factor [19]. Investigations into the epidemiology of KD revealed that individuals living in areas with Se-poor soil were under a high risk of development of the disease. Individuals living in those areas had low dietary intakes of Se that were reflected in low serum and hair levels of Se. Populations living in areas of China with Se-rich soil did not develop KD [25–28].

In this study we have investigated the potential role of Se in anthracycline-induced cardiotoxicity in pediatric cancer patients undergoing chemotherapy. We found an association between low Se levels and anthracycline cardiotoxicity which could be prevented by Se supplementation. These results suggest that Se deficiency may have an effect on anthracycline-induced cardiomyopathy, which may have similarities to KD. 

The family of selenoproteins includes glutathione peroxidases, the redox enzymes that take advantage of the chemical properties of Se to remove free radicals by reduced glutathione and thus to form oxidized glutathione. Se supplementation had a protective effect on ischemia/reperfusion injury in experimental animals; it improved the recovery of cardiac function, decreased ultrastructural changes, increased the expression of glutathione-related enzymes, and partially affected the antioxidant capacity of the tissues together with an effect on gene transcription level [29, 30]. Se supplementation prevented the hypoxia/reoxygenation injury of the isolated neonatal cardiomyocytes and resulted in an NO-related increase of inotropic response of cardiac muscle to the beta-adrenergic stimulation by isoproterenol [17]. Oral Se supplementation has been shown to reverse the biochemical evidence of the Se deficiency [29–31]. The beneficial effect of treatment with the inorganic form of Se was also demonstrated in experimental models of cardiac injury [31, 32]. The mechanism by which Se influences iNOS cardiac expression is unknown. Kim et al. [33] have shown that lipopolysaccharide-activated human T cells with relatively high concentrations of selenite had lower NF-kB-binding and -decreased NO production. Similarly, Turan et al. [34] observed that total NF-kB in the cardiac muscle was reduced by Se. They suggested that Se deficiency or excess affects signal transduction. Se effect can be monitored with Pro-BNP, a good marker of cardiac function [7, 35].

Dietary supplementation of 100 *μ*g Se (sodium selenite) in patients receiving total parenteral nutrition has been reported to prevent arrhythmias and cardiomegaly and lead to an increase in left ventricle ejection fraction [36]. In addition, the incidence of Keshan disease, an endemic dilated congestive myocardiopathy in areas of Se deficiency in China and Russia, has been shown to be decreased by oral Se supplementation at a dosage of 150–300 *μ*g/week [36, 37]. It should be noted that Se supplementation has also been suggested as a strategy for prevention of myocardial disease in other studies of human cardiac pathology [36–38].

The results of our study support the hypothesis that Se supplementation could be considered as a strategy for treatment and prevention of anthracycline-induced cardiomyopathy observed in children with cancer. Our results also suggest that Se supplementation should be continued much longer to ameliorate or prevent anthracycline-induced cardiotoxicity. In conclusion, our results suggest that Se supplementation may have a potential role in the protection against anthracycline-induced cardiac toxicity in patients with high pro-BNP level and/or cardiac failure and low Se levels.

## Figures and Tables

**Table 1:** Selenium and Pro-BNP levels of patients.

	Normal Pro-BNP		High Pro-BNP and/or abnormal	
	(*n* = 56)		ECHO (*n* = 11)	
	Range	Median	Range	Median
*Pro-BNP (pg/mL)	8.2–119.6	32.4	10–8022	226.3
**Selenium (mcg/L)***	51.3–150	99.4	20–129	62

*P<0.001.

**P<0.001.

***n = 26 (twenty-six of fifty-six patients with normal Pro-BNP were measured for Se level) Mann Whitney *U* test was used, and median (range) was given as descriptive statistics.

**Table 2:** Echo findings of patients with high and normal Pro-BNP levels.

	ECHO findings		Total number of patients (%)
	Normal *n* = 29 (%)	Abnormal *n* = 8 (%)	
Normal Pro-BNP levels	25 (96.2)	1 (3.8)	26 (100)
High Pro-BNP levels	4 (36.4)	7 (63.6)	11 (100)

Total	29 (78.4)	8 (21.6)	37 (100)

*P* < 0.001 (Fisher's exact test).

**Table 3:** Se-supplemented patients with low serum Se levels, high Pro-BNP levels, and/or cardiac failure.

Pt. *n* = 11	Total anthracy mg/m^2^	Pro-BNP pg/mL	Se mcg/L	ECHO	Cardiac failure	Digoxin Enalapril Furosem	Se Suppl.	End results				
								Echo	(i) Pro-BNP (pg/mL)	(ii) Pro-BNP (pg/mL)	(i) Se (mcg/L)	(ii) Se (mcg/L)
ES	550	754	70	Systolic failure	+	+	100 mcg	Normal	536	123.5	108	86
NBL	180	175	52	Systolic failure	+	+	100 mcg	Normal	85	95	103	106
ACC	400	10	71	Diastolic failure	−	−	100 mcg	Normal	10	10	65	75
HL	300	843	55	Diastolic failure	−	−	100 mcg	Normal	298	118	72	81
NHL	240	172	49.2	Normal	−	−	100 mcg	Normal	12.6	NA	109	NA
HL	400	197.5	20	Normal	−	−	100 mcg	Normal	80	NA	208	NA
BL	150	170.4	57	Normal	−	−	100 mcg	Normal	2	NA	75	NA
NHL	120	277	83	Systolic failure	+	+	100 mcg	Normal	NA	NA	NA	NA
RMS	0	127	62	Intracardiac thrombus	−	−	—	Normal	67	NA	NA	NA
AML	400	8022	65	Normal	−	−	—	Died	NA	NA	NA	NA
TALL	320	1536	129	Pericardial effusion, tamponade	−	−	—	Died	NA	NA	NA	NA

Pt: patient; NA: not available, ES: Ewing's sarcoma, NBL: neuroblastoma, ACC: adrenocortical carcinoma, HL: Hodgkin lymphoma, NHL: non-Hodgkin lymphoma, BL: Burkitt lymphoma, RMS: rhabdomyosarcoma, AML: acute myeloid leukemia; T ALL: T acute lymphoblastic leukemia.

**Table 4:** Pre- and postsupplementation levels in Se-supplemented patients with low serum Se levels and high Pro-BNP levels and/or cardiac failure.

*n* = 8	Presupplementation levels, range (median)	1st postsupplementation levels, range (median)	2nd postsupplementation levels, range (median)
Pro-BNP (pg/mL)	10–843 (175)	2–536 (73.5)*	10–123.5 (106.5)**
Se (mcg/L)	20–83 (57)	65–109 (103)***	75–106 (83)****

*P = 0.018.

**P = 0.109.

***P = 0.028.

****P = 0.068.

Wilcoxon sign test was used; median and range were given as descriptive statistics.
